# Mutation spectrum, expression profiling, and prognosis evaluation of Fanconi anemia signaling pathway genes for 4259 patients with myelodysplastic syndromes or acute myeloid leukemia

**DOI:** 10.1186/s12920-023-01730-5

**Published:** 2023-11-16

**Authors:** Lixian Chang, Li Zhang, Beibei Zhao, Xuelian Cheng, Yang Wan, Ranran Zhang, Weiping Yuan, Xingjie Gao, Xiaofan Zhu

**Affiliations:** 1 State Key Laboratory of Experimental Hematology, National Clinical Research Center for Blood Diseases, Haihe Laboratory of Cell Ecosystem, Institute of Hematology & Blood Diseases Hospital, Chinese Academy of Medical Sciences & Peking Union Medical College, 288 Nanjing Road, Tianjin, 300020 China; 2 Tianjin Institutes of Health Science, Tianjin, 301600 China; 3 Department of Biochemistry and Molecular Biology, School of Basic Medical Sciences, Excellent Talent Project, The Province and Ministry Co-sponsored Collaborative Innovation Center for Medical Epigenetics, Tianjin Medical University, Heping District Qixiangtai Road No.22, Tianjin, 300070 China

**Keywords:** Fanconi anemia pathway, AML, MDS, Expression, Mutation

## Abstract

**Background:**

Individuals diagnosed with Fanconi anemia (FA), an uncommon disorder characterized by chromosomal instability affecting the FA signaling pathway, exhibit heightened vulnerability to the onset of myelodysplastic syndromes (MDS) or acute myeloid leukemia (AML).

**Methods:**

Herein, we employed diverse bioinformatics and statistical analyses to investigate the potential associations between the expression/mutation patterns of FA pathway genes and MDS/AML.

**Results:**

The study included 4295 samples, comprising 3235 AML and 1024 MDS from our and nine other online cohorts. We investigated the distinct proportion of race, age, French-American-British, and gender factors. Compared to the FA wild-type group, we observed a decrease in the expression of FNACD2, FANCI, and RAD51C in the FA mutation group. The FA mutation group exhibited a more favorable clinical overall survival prognosis. We developed a random forest classifier and a decision tree based on FA gene expression for cytogenetic risk assessment. Furthermore, we created an FA-related Nomogram to predict survival rates in AML patients.

**Conclusions:**

This investigation facilitates a deeper understanding of the functional links between FA and MDS/AML.

**Supplementary Information:**

The online version contains supplementary material available at 10.1186/s12920-023-01730-5.

## Introduction

Fanconi anemia (FA) is a rare autosomal recessive hematological disorder commonly observed in children as a congenital bone marrow failure syndrome. It is closely associated with the FA signaling pathway [1–5]. The FA signaling pathway contains 23 members, such as FANCA, FANCB, and BRCA2 [1–6]. Patients with FA are more prone to developing myelodysplastic syndromes (MDS) and acute myeloid leukemia (AML). Additionally, several other types of tumors may be influenced by genes associated with the FA signaling pathway [7–10].

MDS, a clonal disorder originating from hematopoietic stem cells, has a high propensity for transformation into AML [11–13]. AML involves an aberrant proliferation of primitive and immature myeloid cells in bone marrow and peripheral blood [14–16]. The exact cause of MDS and AML is still unknown, but it is believed to result from genetic and environmental factors. It is speculated that gene mutations and chromosomal abnormalities triggered by biological, chemical, or physical factors contribute to malignant clonal proliferation [11–16]. The FA signaling pathway is crucial for preserving genetic stability when cells are stressed [8, 9, 17]. However, the deeper connection between the expression/mutation pattern of FA signaling pathway members and clinical MDS and AML diseases is not yet fully understood.

In this study, we conducted a comprehensive investigation by combining the cohort in our center with the other nine cohorts. Utilizing available gene expression, mutation, and corresponding clinical traits, we analyzed various aspects of MDS/AML patients, including the FA mutation spectrum, expression profile, prognosis evaluation, cytogenetic risk classifier, and nomogram prediction model. This was achieved through various approaches, such as batch correction, principal component analysis (PCA), correlation analysis, nomograms, and the random forest/decision tree.

## Methods

### Included cohorts

Our study included 10 cohorts with a total of 4295 samples. The cohorts were named as follows: SELF_FA (*n* = 13), TCGA-LAML (*n* = 137), TARGET-AML (*n* = 291), BeatAML (*n* = 650), MDS_mskcc_2020 (*n* = 858), Papaemmanuil_MDS_2013 (*n* = 573), Papaemmanuil_NEJM_2016 (*n* = 1319), LAML-CN (*n* = 77), LAML-KR (*n* = 205), and WASHU (*n* = 136). To display the bar graph of each cohort, we used the **“**ggbarplot” R package. Additionally, we obtained a hollow pie chart to represent the proportion of MDS and AML using the **“**ggplot2**”** and **“**ggforce” R packages. The SELF_FA cohort specifically included mutation data from 13 FA patients who developed MDS or AML. The mutation data of the other cohorts was obtained from the cBioPortal database [18–21]. Three cohorts (TCGA-LAML, TARGET-AML, BeatAML) contained the gene expression matrix. Data on gene expression and clinical outcomes were downloaded from the TCGA-LAML cohort using a “TCGAbiolinks” R package. The data on TARGET-AML and related clinical traits were obtained from the official website (https://ocg.cancer.gov/). The expression and clinical characteristics within the BeatAML cohort were obtained from the supplementary materials of the publication [22]. Clinical data were obtained from the cBioPortal database [18–21].

To visualize the proportion of AML and MDS patients based on gender, race, cytogenetic risk, and FAB, we employed the “plotrix” R package. Additionally, we conducted the Wilcox test to examine the tumor mutational burden (TMB) among AML or MDS patients. We presented the findings using the “ggbarplot” function from the “ggpubr” R package.

### Genetic mutation analysis

The specific mutation sites of FA pathway genes were illustrated in the protein structure of patients with AML or MDS. The mutation number and percentage of FA pathway genes in the patients of AML and MDS were visualized using a “ggplot2” R package. Additionally, we utilized the “pheatmap” R package to display the relationship between the mutation, expression, and related clinical traits of the FA pathway gene. FLT3, FA, CEBPA, IDH1, NPM1, and specific FA pathway gene mutation rates in AML and MDS patients were further analyzed and presented as a radar diagram by an “fmsb” R package. We also compared the differences in race, age, FAB, gender, type, and cytogenetic risk factors between the FA wild-type and mutation groups. The “plotrix” R package was used to visualize the data. Furthermore, the Wilcox test was performed to analyze the difference in age index, and the “ggbarplot” function was utilized for the visualization. Finally, we analyzed the mutation percentages of IDH1, NPM1, CEBPA, and FLT3 between the FA wild-type and mutation groups, and these results were visualized using the **“**barplot” function.

### Expression analysis

The expression matrices of TCGA-LAML, TARGET-AML, and BeatAML datasets were processed using the R language. Batch correction was performed using the “sva” R package. PCA was conducted using the “prcomp ()” function, and the effect before and after the correction was visualized using the “ggplot2” R package. To examine the expression pattern of FA pathway genes in different FAB groups, the “ggballoonplot” function was used.

Based on the corrected matrix, the difference in the expression of each FA gene between the wild-type and overall mutant-type of FA pathway genes was analyzed using a Wilcox test and “ggplot2” R package. Also, a Wilcox test was performed to determine the correlation between mutation and expression of specific FA pathway genes, including XRCC2, BRCA1, FANCE, FAAP100, FANCC, and MAD2L2. Visualization was performed using the “ggviolin” function.

We performed a Spearman correlation analysis using the “cor.Test” function to explore the links of the FA pathway gene expression with TMB. We visualized the correlation between TMB and expression of each FA gene as a radar diagram using an “fmsb” R package. We displayed the correlation among genes using a “Corrplot” R package as a heat map. Additionally, we utilized the “iGraph” R package to create a network visualization in the form of a tree layout. Finally, we generated scatter plots using the “ggplot2” R package to analyze the correlation between TMB and the hub gene expression within the network, such as BRIP1, FANCE, ERCC4, and MAD2L.

### Prognosis evaluation

We utilized the “survival” and “survminer” packages to evaluate the overall survival (OS) prognosis of patients with MDS + AML, MDS, and AML. We focused on the wild-type and overall mutant FA pathway genes, specifically FANCA. The subgroup survival analyses took into consideration factors such as cytogenetic risk, TMB, FLT3 mutation, and NPM1 mutation. To conduct the OS prognosis analysis, we used the expression matrix of the FA gene and employed the “surv_cutpoint” function to select the optimal cutoff. In addition, we performed receiver operating characteristic curve (ROC) analyses to assess the OS prognosis of TARGET-AML at 1-, 3-, and 5-year survival time points. The area under the ROC curve (AUC) value was calculated.

### Nomogram prediction model

A cph () modeling analysis was conducted on the training set using a “regplot” R package. Subsequently, a Nomogram was generated. The calibration curves were obtained using the calibrate () function of “ggstatsplot”. The “cph”, “validate”, “predict” functions of “rms” were used to yield the C_index along with a 95% confidence interval (CI).

### Random forest and decision tree

Cytogenetic risk was used as the outcome variable in our study. We utilized the “randomforest” R package to conduct the random forest analysis, which provided us with mean decrease Gini and mean decrease accuracy values. We employed the “ggdotchart” function to visualize the results. Further, we performed ROC analysis using the “pROC” R package. For our decision tree analysis, the samples were randomly divided into training and testing sets (2/3 and 1/3, respectively). We used the “rpart” R package for this analysis and visualized the decision tree using the “rpart Plot “R package.

## Result

### Analytic strategy

**Fig. 1:**
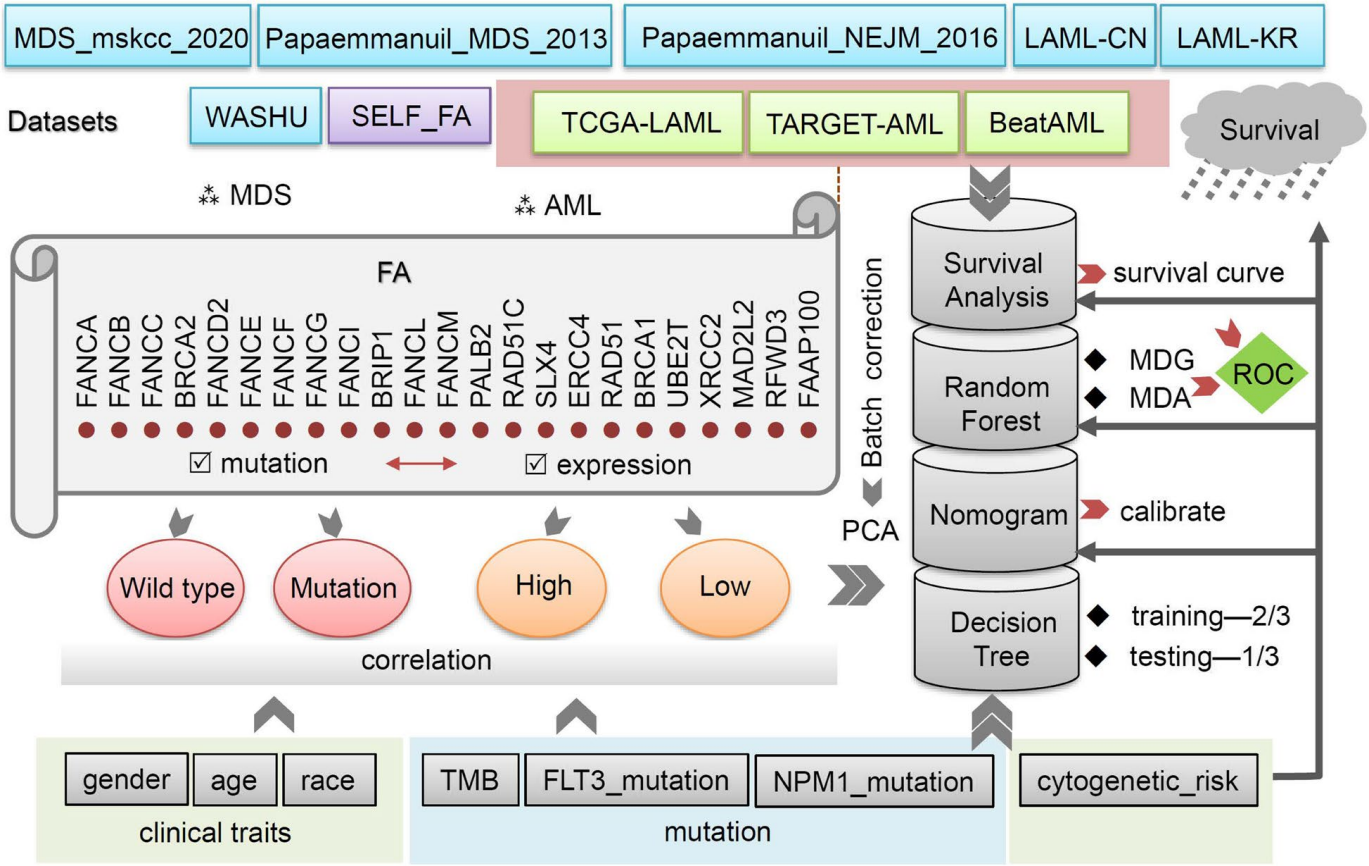
Analysis strategy of this study

Figure 1 illustrates our analytic strategy. We included 10 cohorts consisting of MDS and AML patients, namely TCGA-LAML, TARGET-AML, BeatAML, MDS_mskcc_2020, Papaemmanuil_MDS_2013, Papaemmanuil_NEJM_2016, LAML-CN, LAML-KR, and WASHU. Within TCGA-LAML, TARGET-AML, and BeatAML cohorts, we performed batch correction of the expression matrix and assessed the treatment effect through PCA. We then extracted the expression and mutation data of 23 FA pathway genes and conducted correlation analysis. Patients were divided into wild-type (Wt) and overall mutant-type (Mut) groups based on the overall mutation status of FA genes. Additionally, based on FA gene expression levels, we obtained high- and low-expression groups. We conducted the correlation analyses with clinical traits such as gender, age, race, cytogenetic risk, and TMB, FLT3, and NPM1 mutation. Next, after integrating survival status and time, we conducted the KM survival curve analyses. We also performed random forest analysis to obtain the values of MDG and MDA and built a decision tree using the training (2/3 samples) and testing (1/3 samples) set. ROC analyses were utilized to evaluate the effect. Finally, we plotted a nomogram and evaluated the corresponding calibration curve effect.

### Basic characteristics of the cohorts

**Fig. 2:**
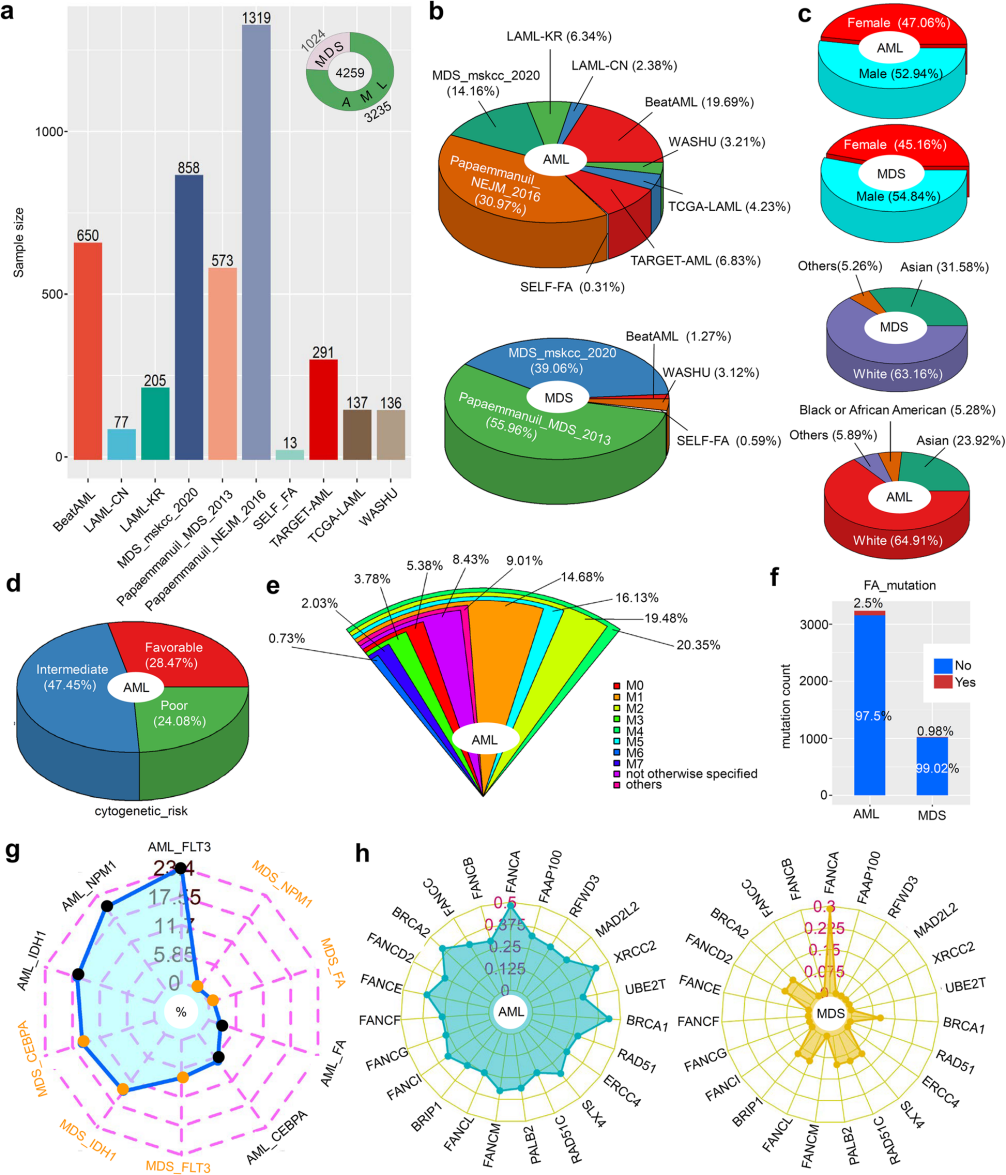
Basic FA expression and mutation characteristics in patients with MDS/AML. A total of 4259 cases from 10 cohorts were included in the study (**a**). The bar graph of each cohort was displayed using the “ggbarplot” R package. The proportion of MDS and AML was represented in a hollow pie chart using the “ggplot2” and “ggforce” R packages. The “plotrix” R package was used to visualize the proportion of AML and MDS patients in different groups of cohorts (**b**), gender, and race (**c**). In contrast, a “plotrix “R package was for the cytogenetic risk (**d**) and FAB (**e**) of AML patients. The mutation number and percentage of FA pathway genes in AML and MDS patients were analyzed, and the results were visualized using a “ggplot2” R package (**f**). We showed the mutation rates of FLT3, FA, CEBPA, IDH1, NPM1 (**g**), and FA pathway genes (**h**) in AML and MDS patients through radar diagrams created with the “fmsb” R package

**Fig. 3:**
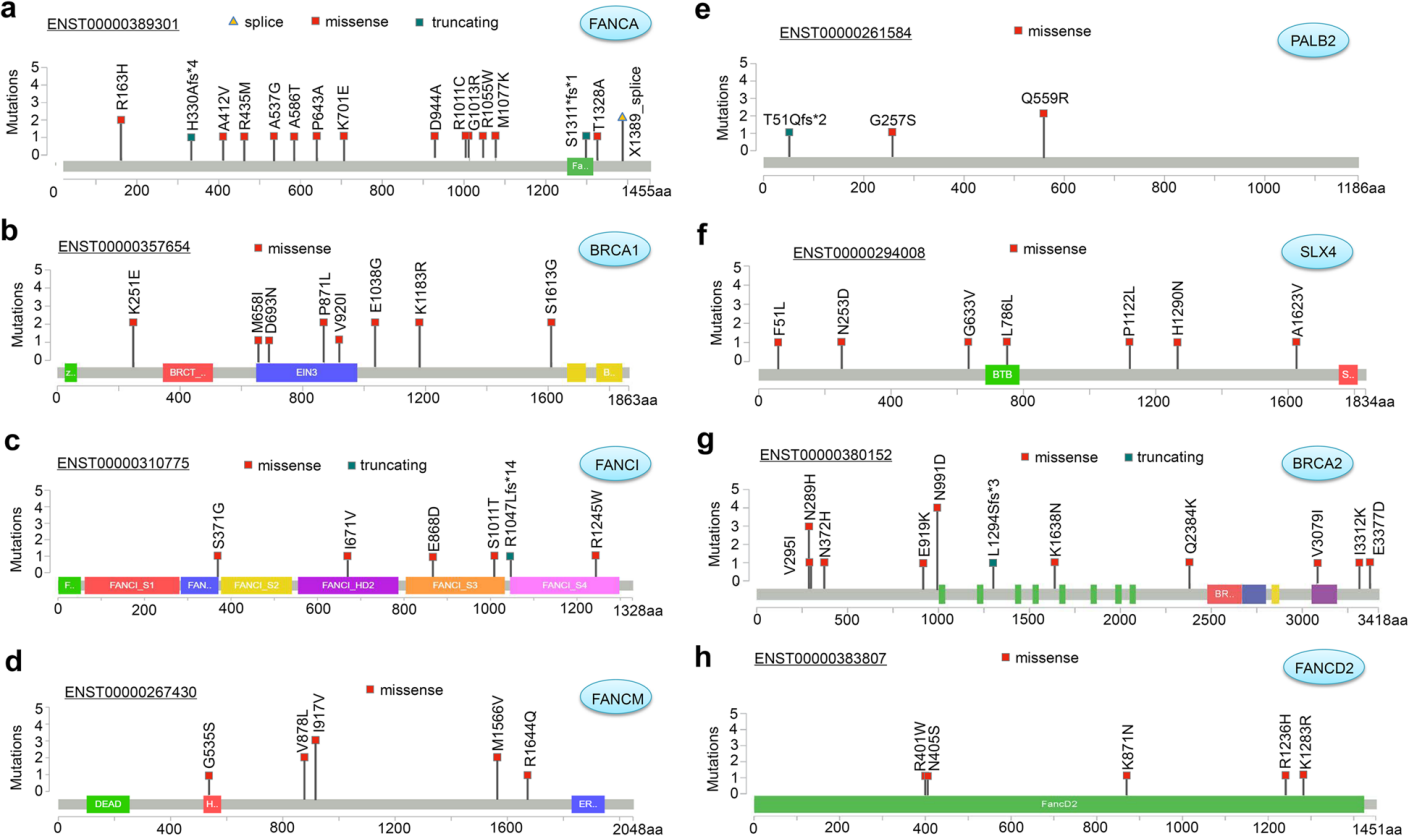
Mutation features of FA pathway genes with relatively high mutation frequency in cases of MDS/AML. (**a**) FANCA; (**b**) BRCA1; (**c**) FANCI; (**d**) FANCM; (**e**) PALB2; (**f**) SLX4; (**g**) BRCA2; (**h**) FANCD2

Figure 2a displays the sample size of each cohort included in our study. A total of 4295 samples were analyzed, with 3235 samples being AML and 1024 samples being MDS. Then, we analyzed the proportion of AML and MDS patients in each cohort in depicted Fig. 2b. When considering gender and race, we observe a slightly higher proportion of male patients with AML or MDS compared to female patients, and the majority of the samples were from the white population (Fig. 2c). The cytogenetic risk proportion of AML samples revealed that 47.45% were intermediate, 24.08% were poor, and 28.47% were favorable (Fig. 2d). Furthermore, the analysis of FAB proportion (Fig. 2e) demonstrated that the M4 subtype had the highest representation among AML samples (20.35%). Subsequently, we investigated the number and proportion of mutations in the FA pathway genes in patients with AML and MDS. AML patients had a higher frequency of FA gene mutations than MDS patients (2.5% > 0.98%), as shown in Fig. 2f. The radar diagram in Fig. 2g further illustrates the overall mutation rate of FLT3, NPM1, LDH1, CEBPA and FA gene in AML and MDS patients. Among them, FLT3 had the highest mutation rate in AML patients (23.4%), followed by NPM1 with a mutation rate of 20.4% in AML patients. Additionally, we presented the mutation rate of each FA pathway gene in Fig. 2h. Mutations in FANCA were the most prevalent (0.46%), followed by BRCA1 (0.37%) and XRCC2 (0.34%) in AML patients, while FANCA mutation rate was the highest in MDS patients (0.29%). Figure 3 and Fig. S1 displayed the specific mutation sites of each FA pathway member. Missense mutations were the most common type among MDS/AML cases, with mutations such as R163H, A412V, R435M, and A537G identified in FANCA (Fig. 3a). The N991D mutation of BRCA2 was detected in four samples (Fig. 3g).

### Basic characteristics and FA mutations

**Fig. 4:**
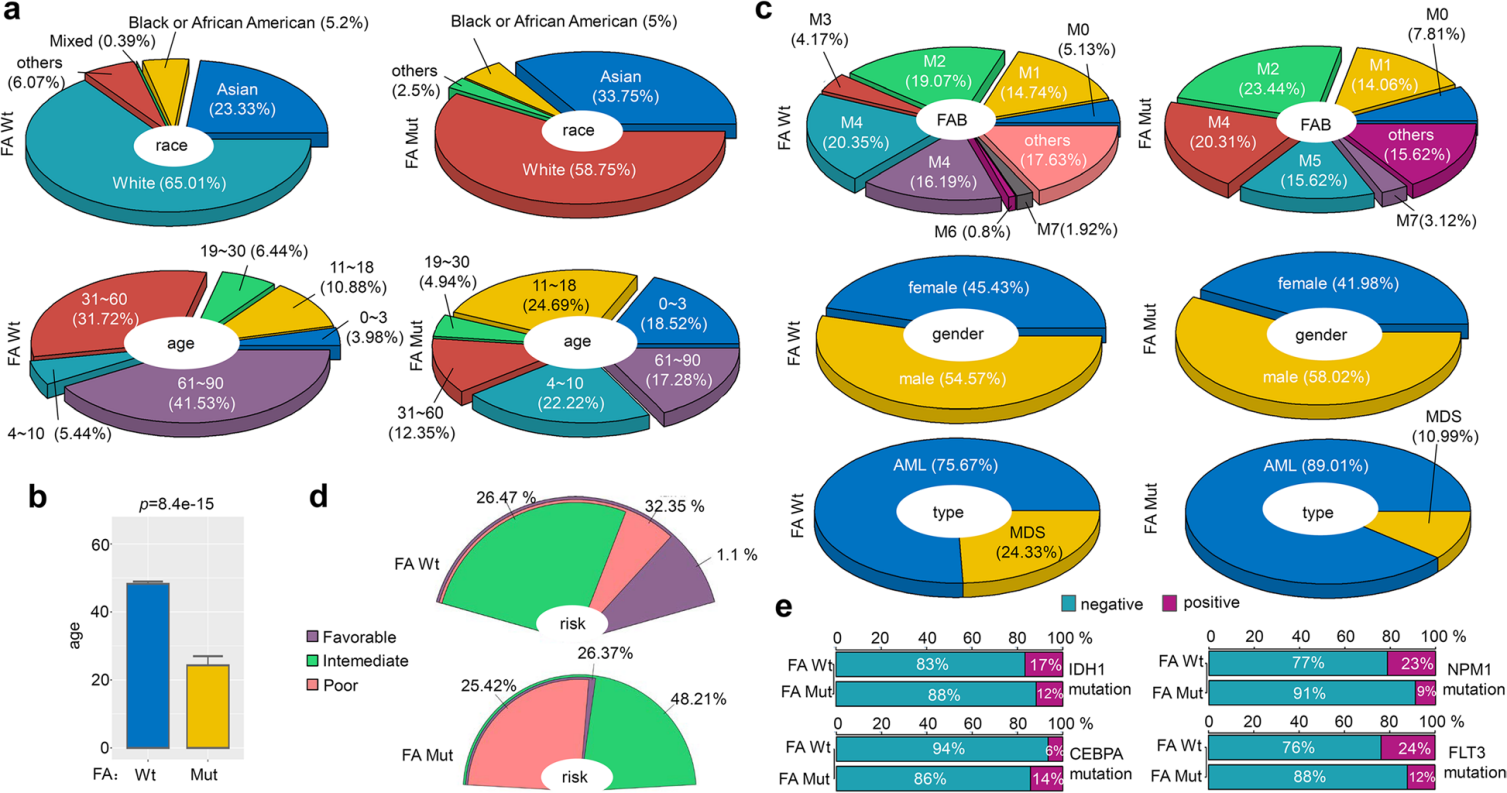
Basic characteristic difference between FA wild-type and mutant-type groups. We analyzed the differences in the factors, including race, age (**a-b**), FAB, gender, type (**c**), and cytogenetic risk (**d**) between the wild-type (Wt) and overall mutant-type (Mut) of FA pathway genes. The results were visualized using the “plotrix” R package. We also conducted the Wilcox test to analyze the difference in age index, and the results were visualized using the “ggbarplot” function of the “ggpubr” R package (**b**). We then analyzed the mutation percentages of IDH1, NPM1, CEBPA, and FLT3 between the Wt and Mut groups of FA, and the results were visualized using the “barplot” function (**e**)

Next, we analyzed various factors, including race, age, FAB, gender, type, cytogenetic risk, and mutations in IDH1, NPM1, CEBPA, and FLT3 genes, based on overall mutations in FA pathway genes. Figure 4a illustrates that in comparison to the FA wild-type (Wt) group, there was a decrease in the proportion of individuals classified as “White (race)” (from 65.01 to 58.75%), while there was an increase in the proportion of individuals in the “Asian (race)” category (from 23.33 to 33.75%), within the FA overall mutant-type (Mut) group. Regarding age distribution, most individuals in the FA Wt group were found within the 61 ~ 90 years category (Fig. 4a, 41.53%). In contrast, the highest proportion of individuals in the FA Mut group fell within the 11–18 age category (24.69%). Also, the FA Mut group exhibited a statistically significant lower mean age compared to the FA Wt group (Fig. 4b, *p* = 8.4e-15). Analysis of FAB classification revealed that the individuals in the FA Wt group has the highest proportion of M4 subtypes (20.35%), while cases in the FA Mut group were more likely to be M2 carriers (23.44%). In comparison to the FA Wt group, there was a slight rise in the percentage of individuals identified as “male (gender)” (Fig. 4c, from 54.57 to 58.02%) and an increase in the percentage of cases diagnosed with “AML (type)” (Fig. 4c, from 75.67 to 89.01%), and “intermediate (cytogenetic risk)” (Fig. 4d, from 26.47 to 48.21%) in the FA Mut group. Finally, we explored the correlation between FA mutation and other mutations in IDH1, NPM1, CEBPA, and FLT3 genes. As depicted in Fig. 4e, the FA Mut group exhibited a lower mutation rate of IDH1, NPM1, and FLT3 but a relatively higher mutation rate of CEBPA compared to the FA Wt group.

### Correlation between FA gene expression and mutation

**Fig. 5:**
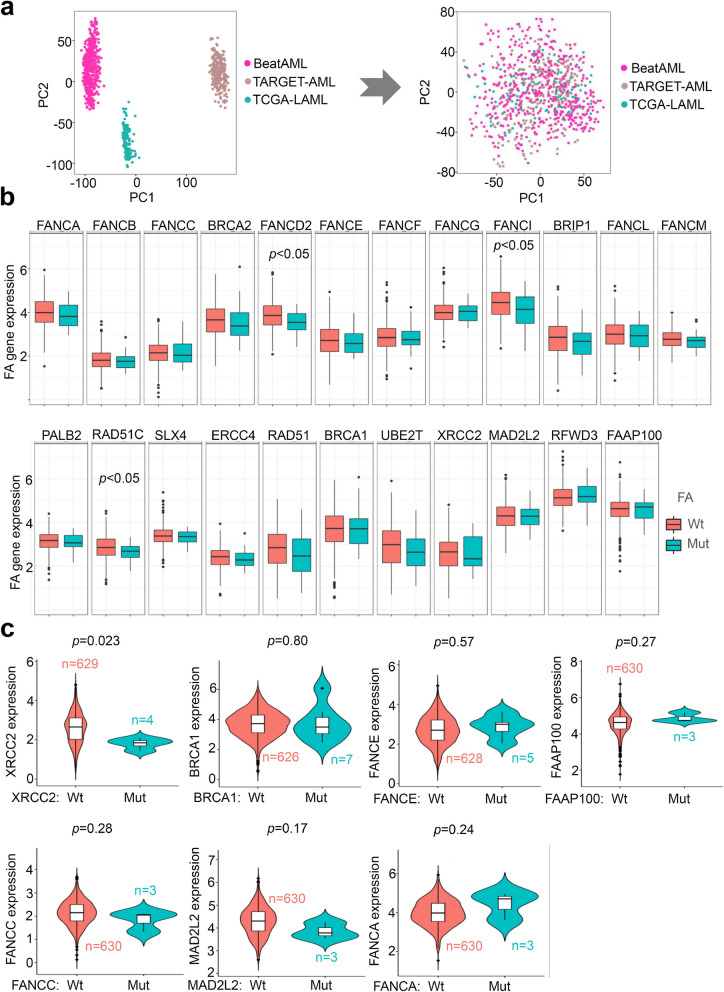
Correlation between FA gene expression and mutation. We utilized the “SVA” R package to calibrate gene expression matrices within the BeatAML, TARGET-AML, and TCGA-LAML cohorts. We then conducted a PCA analysis using the “prcomp” function and “ggplot2” (**a**). Next, we analyzed the expression difference of each FA gene between the wild-type (Wt) and overall mutant-type (Mut) of FA pathway genes using the Wilcox test and “ggplot2” R package (**b**). We also performed the Wilcox test to examine the correlation between mutation and expression of specific FA pathway genes, such as XRCC2, BRCA1, FANCE, FAAP100, FANCC, MAD2L2, and FANCA. The results were visualized using the “ggviolin” function from the “ggpubr” package (**c**)

We first conducted batch calibration of gene expression matrices within BeatAML, TARGET-AML, and TCGA-LAML cohorts. Subsequently, a PCA analysis was conducted to assess the removal of batch differences among these cohorts (Fig. 5a). Furthermore, we investigated the correlation between mutation and expression of FA genes, along with related clinical traits, using a heat map (Fig. S2). The FA pathway gene expression patterns in different FAB classifications were also depicted in Fig. S3. Next, we compared the statistical differences in FA gene expression between FA Wt and Mut groups. As depicted in Fig. 5b, we observed the downregulation of FNACD2, FANCI, and RAD51C in the FA Mut group when compared with the FA Wt group (*p* < 0.05). Further, we examined the correlation between specific FA gene expression and mutation. Notably, when XRCC2 was mutated, its expression level decreased (Fig. 5c, *p* = 0.023), although this correlation was not observed in other FA genes.

**Fig. 6:**
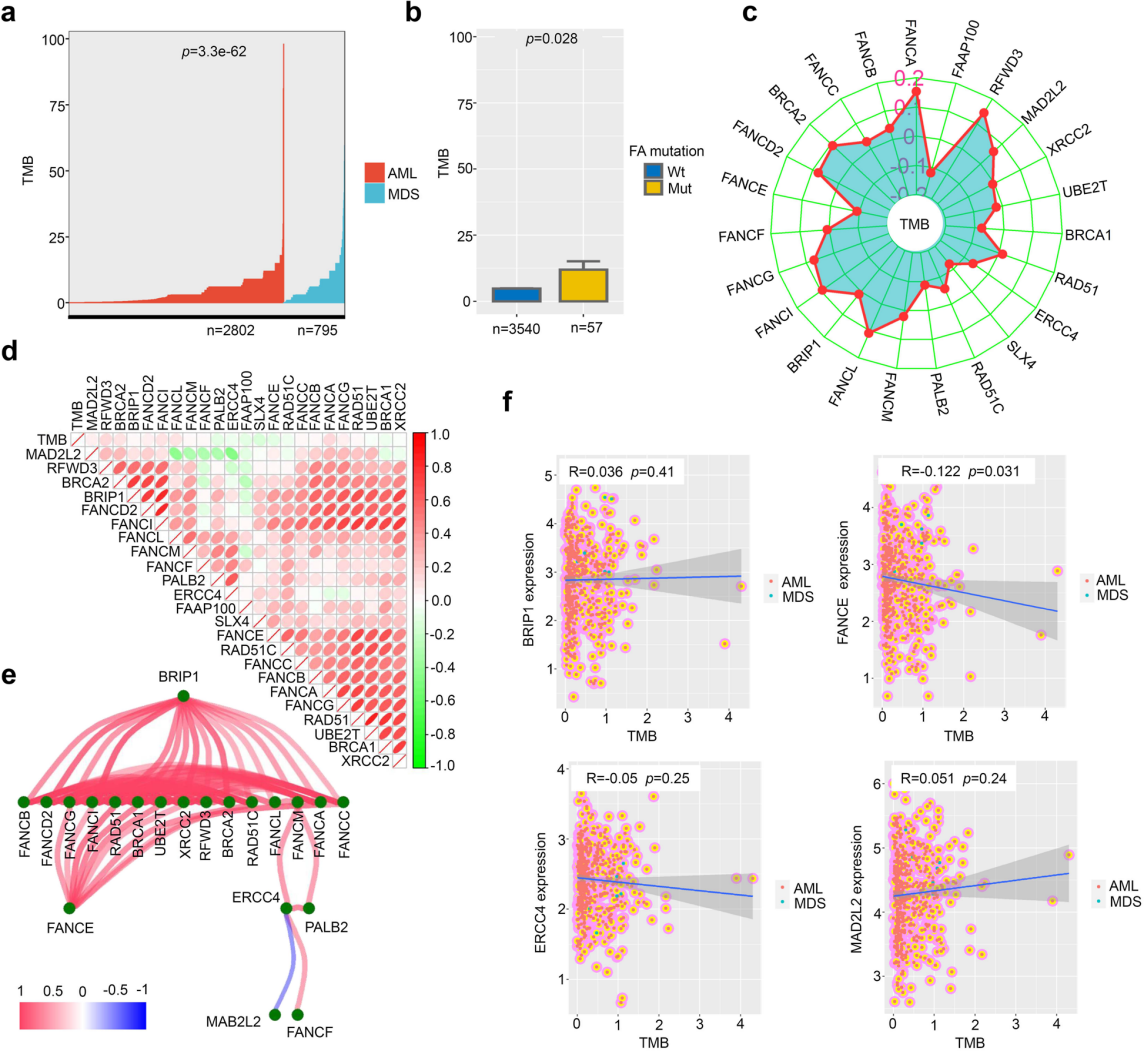
Relationship between FA gene expression and TMB. Firstly, we employed the Wilcox test to analyze the TMB of AML and MDS patients, and the results were displayed using the “ggbarpAlot” function (**a-b**). We also used the cor.test to examine the Spearman correlation between the gene expression values of FA pathways and TMB. To visually represent the correlation between TMB and the expression of each FA gene, we created a radar diagram using the “fmsb” R package (**c**). Furthermore, a heat map was generated using the “Corrplot” R package to display the relationships among genes (**d**). To provide a network visualization in the form of a tree layout, we utilized the “iGraph” R package (**e**). Finally, scatter plots illustrating the correlation between TMB and the expression of hub genes within the network, namely BRIP1, FANCE, ERCC4, and MAD2L2, were created using the “ggplot2” R package (**f**)

Next, we examine the relationship between FA gene expression/mutation and TMB. The TMB profile of AML and MDS patients who participated in this study is presented in Fig. 6a. Our findings revealed a higher TMB value in the FA Mut group compared to the FA Wt group (Fig. 6b, *p* = 0.028). To further explore this relationship, we depicted the correlation between TMB value and FA gene expression using a radar diagram (Fig. 6c). Notably, TMB expression was positively correlated with FANCA expression (Fig. 6c, *p* < 0.01, *R* = 0.153).

The expression correlation between different FA genes was analyzed using a heat map in Fig. 6d. Most FA genes showed a positive correlation, except MAD2L2, which was negatively correlated with ERCC4, FANCL, and FANCM. A network diagram in the form of “layout_AS_tree” (Fig. 6e) was presented to illustrate the relationship further. It was observed that BRIP1 and FANCE could serve as the hub genes, while MAB2L2 showed a negative correlation with ERCC4. Finally, a correlation analysis was performed for AML and MDS patients, focusing on BRIP1, FANCE, ERCC4, MAD2L2, and TMB. In Fig. 6f, a negative correlation between FANCE and TMB was observed (*p* = 0.031, *R* = − 0.122), although it was not statistically significant in the other groups.

### Prognostic analysis of FA gene expression and mutation

Here, we investigated the association between FA gene expression, mutation, and the clinical prognosis of MDS and AML patients. Our findings, as shown in Fig. S4, revealed a significant association between a poor OS prognosis and the high expression of several genes, including FANCB (*p* = 0.002), BRCA2 (*p* = 0.013), FANCD2 (*p* = 0.003), FANCE (*p* = 0.015), FANCI (*p* = 0.029), BRIP1 (*p* = 0.004), FANCL (*p* = 0.005), FANCM (*p* = 5.46e-04), PALB2 (*p* = 0.004), RAD51C (*p* = 0.002), SLX4 (*p* = 0.002), ERCC4 (*p* = 0.002), RAD51 (*p* = 0.004), BRCA1 (*p* = 0.003), UBE2T (*p* = 0.014), XRCC2 (*p* = 0.007), and MAD2L2 (*p* = 6.67e-04) genes. Conversely, we observed that low expression of FANCG (*p* = 0.011), RFWD3 (*p* = 0.043), and FAAP100 (*p* = 0.01) genes was associated with a poor prognosis.

**Fig. 7:**
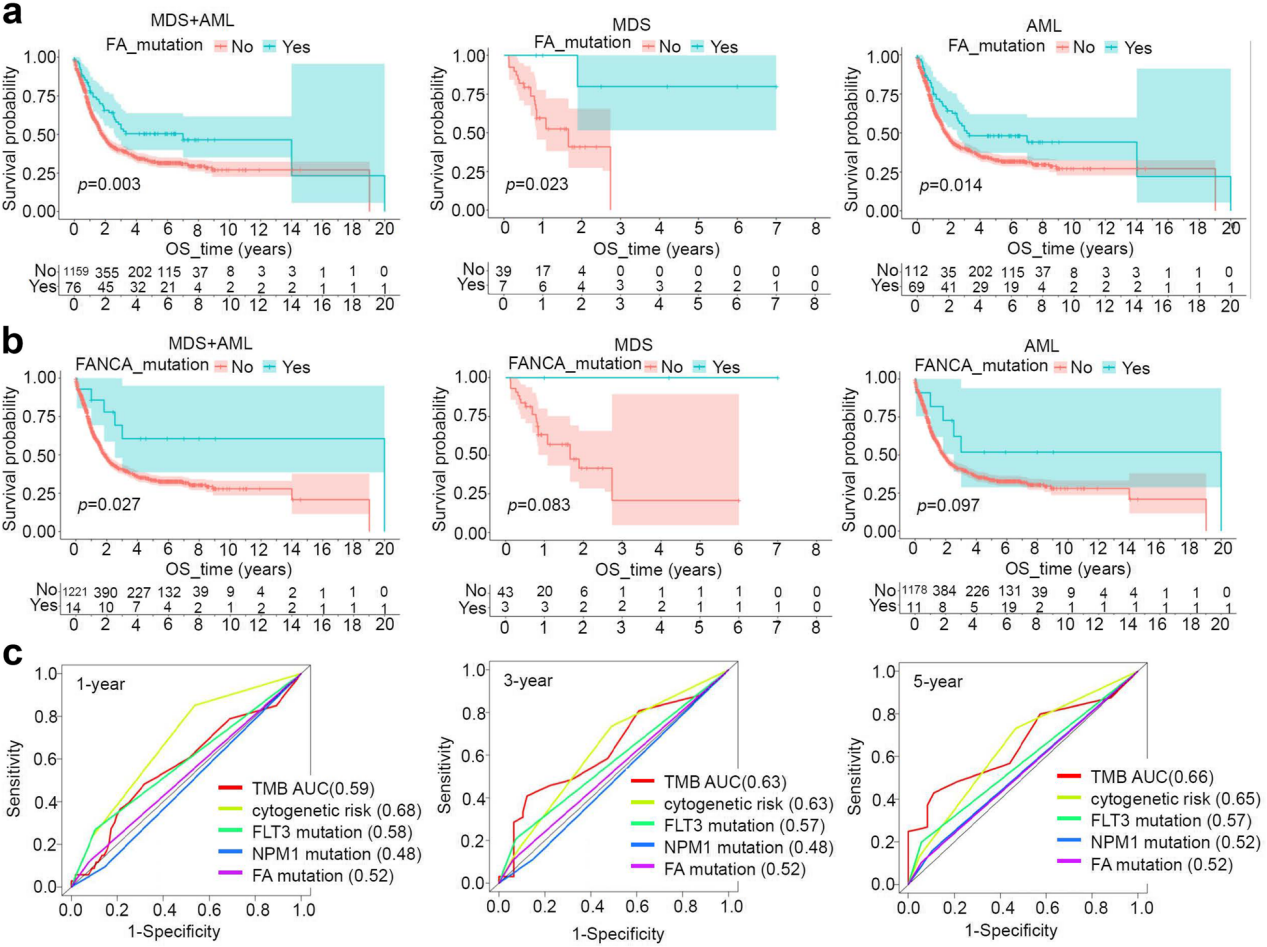
Prognosis evaluation of FA mutation. We utilized the “survival” and “surviviner” packages to assess the OS prognosis of MDS + AML, MDS, and AML patients. The evaluation was conducted for both the wild-type (Wt) and overall mutant-type (Mut) of FA pathway genes (**a**), as well as for the Wt and Mut groups of FANCA (**b**), Targeting the factors (TMB, cytogenetic risk, FLT3/NPM1/FA mutation), ROC analyses were performed, and the AUC values for the OS prognosis were calculated for the 1-, 3-, and 5-year survival time points (**c**)

Subsequently, the correlation between the overall mutation rate of FA pathway genes and the prognosis of OS was analyzed. In Fig. 7a, the FA Mut group had a better clinical OS prognosis for MDS + AML (*p* = 0.003), MDS (*p* = 0.023), and AML (*p* = 0.014) patients. Specifically, it was observed that MDS + AML patients with mutant FANCA also exhibited a better clinical OS prognosis (Fig. 7b, *p* = 0.027). Although there was no statistical difference, MDS and AML patients with mutant FANCA also showed a tendency towards a better prognosis of clinical OS (Fig. 7b). As for cytogenetic risk, AML patients in the intermediate and poor group had a poorer prognosis compared to the favorable risk group (Fig. S5a, *p* = 0.003). However, the intermediate and poor risk groups did not differ significantly (Fig. S5b-d).

Then, we conducted further subgroup prognostic analysis of cytogenetic risk using the FA mutation factor. However, we found no statistical correlation between FA mutation and OS prognosis in the favorable, intermediate, and poor cytogenetic risk subgroups. Besides, we observed that MDS + AML (Fig. S6a, *p* = 5.0e-05), MDS (Fig. S6b, *p* = 0.036), and AML (Fig. S6c, *p* = 7.2e-05) patients with high TMB were associated with a poor prognosis of clinical OS. When we combined the FA mutation and TMB factors, the patients with high TMB but without FA mutation showed a poor OS prognosis (Fig. S6d, *p* = 0.014). However, no statistical difference was detected in the subgroup of FA mutation (Fig. S6d, *p* = 0.413). Additionally, we observed that MDS + AML (Fig. S7a, *p* = 1.8e-04) and AML patients (Fig. S7b, *p* = 1.2e-04) with the FLT3 mutation showed a poor prognosis, compared to the FLT3 wild-type group. Furthermore, we obtained a similar result in the subgroup analysis of AML patients without FA mutation (Fig. S7c, *p* < 0.001). NPM1 mutations were slightly related to OS risk in patients with AML (Fig. S8a, *p* = 0.046), but no significant correlation in other analyses (Fig. S8b-c).

We assessed the survival prognosis of TMB, cytogenetic risk, FLT3, NPM1 mutation, and FA mutation for 1, 3, and 5 years, respectively. We then plotted the ROC curve with the AUC value. Figure 7c demonstrates that the AUC value of the cytogenetic risk index for 1, 3, and 5 years of survival prognosis was more significant than 0.6, indicating a good evaluation effect. The AUC value of the FA mutation index for survival prognosis is better than that of NPM1 but inferior to that of FLT3.

### Nomogram and related assessments

**Fig. 8:**
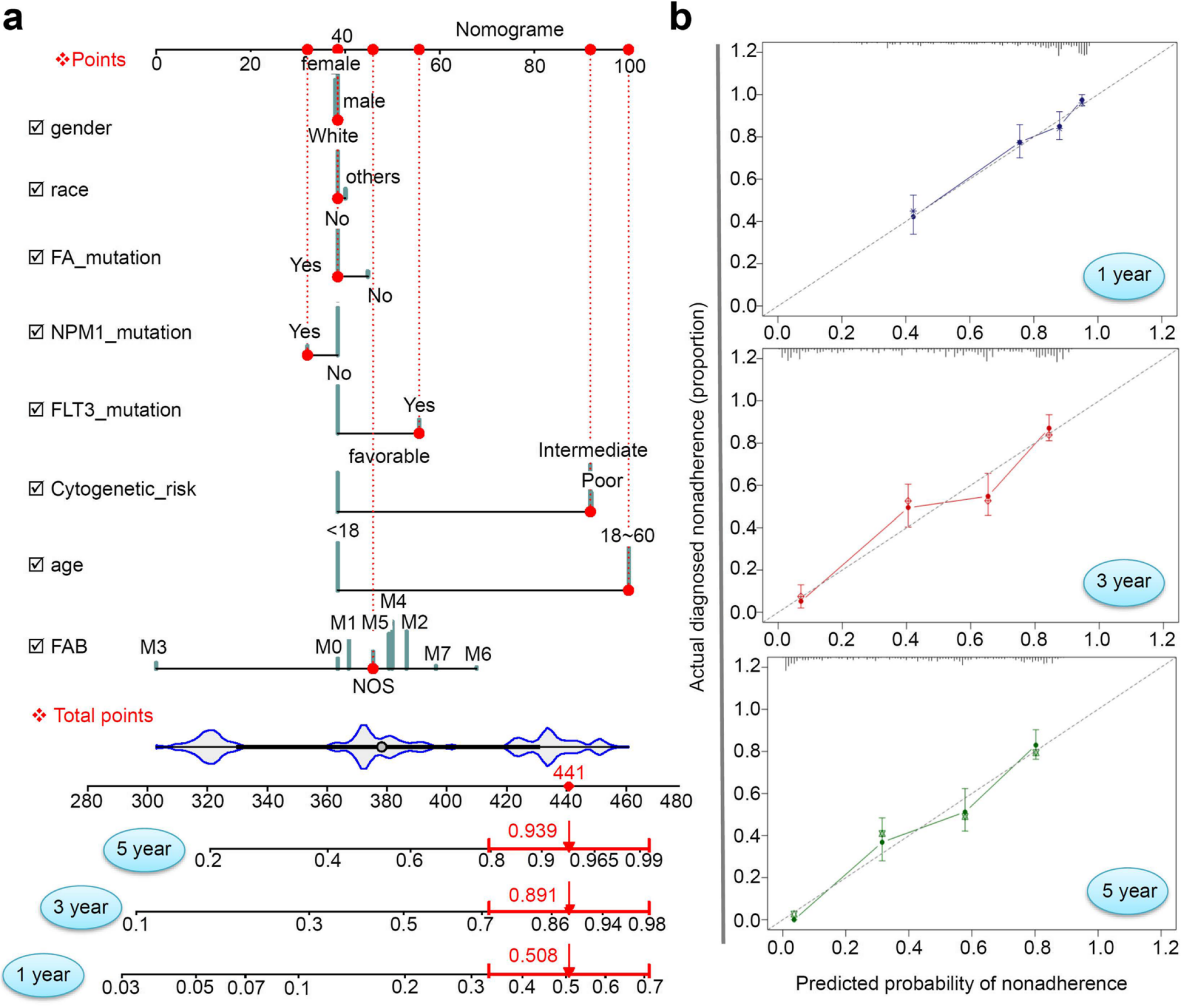
Nomogram and related assessment analyses. To assess the impact of factors such as gender, race, age, FAB, FA/NPM1/FA mutation, or cytogenetic risk, we utilized the “rms” and “regplot” R packages to generate a Nomogram for predicting the 1-, 3-, and 5-year survival rates of a given AML case within the TCGA-LAML cohort (**a**). The calibration plot curves were obtained by employing the “calibrate” function from the “ggstatsplot” R package (**b**)

Next, we conducted the nomogram plotting and related assessments to predict the overall survival rates of AML patients. We combined the factors of gender, race, age, FAB, FA/NPM1/FLT3 mutation, and cytogenetic risk to develop a nomogram. In Fig. 8a, we present the predicted 1-, 3-, and 5-year survival rates as 0.508, 0.891, and 0.939, respectively. We obtained a C index value of 0.765 (95% CI: 0.736 ~ 0.794). The calibration plot curve in Fig. 8b shows a high overlap between the predicted and observed value lines.

### FA-related random forest and decision tress analysis

**Fig. 9:**
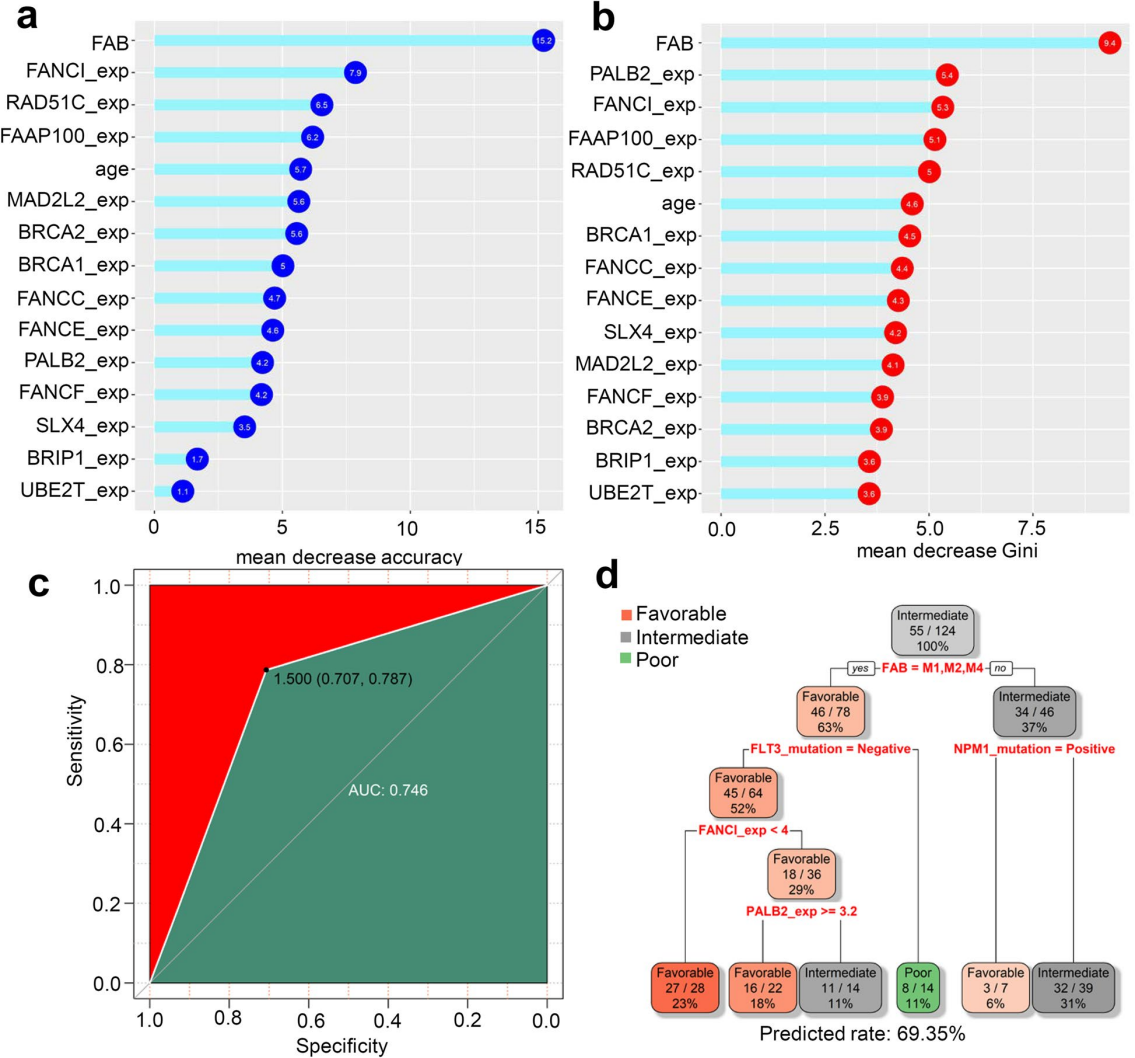
Random forest and decision tree analysis. Based on the expression value of each FA gene and related clinical traits, we utilized the cytogenetic risk as the outcome variable. We obtained the values of mean decrease accuracy (**a**) and mean decrease Gini (**b**) for random forest analysis using a “randomforest” R package. The result was visualized using the “ggdotchart” function. Subsequently, we performed ROC analysis with the “pROC” R package (**c**). For the decision tree analysis, we randomly selected 2/3 samples as the training set and 1/3 as the testing set and visualized the result using the “rpart” R package (**d**)

Finally, considering the strong correlation between cytogenetic risk and the clinical prognosis of AML, we chose cytogenetic risk as the outcome variable for our random forest analysis. We analyzed each FA gene’s expression value and relevant clinical traits. Our results, shown in Fig. 9a-b, demonstrated that classifiers incorporating factors such as FAB, age, FANCI, FAAP100 expression, and others contribute to cytogenetic risk classification in AML patients. The ROC analysis yielded high evaluation effectiveness with an AUC of 0.746 (Fig. 9c). Additionally, using FAB, FLT3 mutation, NPM1 mutation, FANCI, and PABL2 gene expression as predictors, we constructed a decision tree (Fig. 9d) that achieved a predicted classification rate of 69.35% for the factor of cytogenetic risk.

## Discussion

There is a link between Fanconi anemia and a variety of tumor types, including AML [23, 24]. The functional loss of FA pathway during the DNA repair process is crucial for tumor occurrence and development [8, 9, 17, 23]. Extensive research has been conducted to understand the molecular mechanism behind this phenomenon. Our study involved the SELF_FA cohort at our center, which consisted of 13 FA patients with mutations in the FANCA, FANCE, FANCL, FANCM, SLX4, and FANCD2 genes. Among them, six cases developed into MDS and seven into AML. Previous studies have detected FA protein profile abnormalities in 10 cell lines of AML and primary cells from 15 AML patients [25]. Our study combined our cohort with nine additional cohorts to investigate the potential functional involvement of FA pathway gene expression and mutations in the clinical traits of patients with MDS (*n* = 1024) and AML (*n* = 3235) using various research strategies.

In the FAB classification, seven subtypes of AML (M0-M7) have been identified, each with distinct pathogenesis and treatment response [26, 27]. Among 3235 AML cases analyzed, the M4 subtype (acute myelomonocytic leukemia) accounted for the highest proportion, followed by M2 (acute myelomonocytic leukemia with matching) and M5 (acute monocytic leukemia) subtypes. In our cohort, the M5 subtype had a higher proportion of AML cases from FA. Abnormal FANCA function has been associated with the clonal progression of AML [28]. In certain sporadic AML cases, gene deletions and reduced expression of FANCA are linked to the promotion of genetic instability [29]. AML patients have a slightly higher mutation rate of the FA gene than MDS patients (2.5% vs. 0.98%), with FANCA mutation being the most common, consistent with our center’s data. This may be attributed to FANCA, FA’s most frequently mutated gene. Among the 13 cases in our center, five had FANCA mutations, including R1055W, H330Afs*A, K701E, R435M, R1011C, D944A, BRCA2 L1294Sfs*3, and c.2165 + 10A > T splicing variant. Further analysis revealed that MDS/AML patients with mutant FANCA had a better clinical overall survival prognosis than those with wild-type FANCA. Regarding FANCA gene expression, we observed a trend toward better prognosis in MDS/AML patients with high FANCA expression, although statistical significance was not reached. Further, A slightly increased expression of FANCA was noted in the FANCA mutation group, but it was not statistically significant, possibly due to the limited number of mutations.

In addition to FANCA gene mutation, we also detected the mutations in FANCE (R371W), FANCL (C334R, R231C), FANCM (I917V, R1644Q), SLX4 (A1623V, H1290N, F782L, F51L), and FANCD2 (R1236H) in the cases of our center. As evaluated by the KM survival curves, high expression of FANCE was related to a poor OS prognosis, but no statistical link emerged between FANCE mutation and OS. Furthermore, the mutation rate of these genes was not high among the 4259 subjects included in this study, and there were only a few patients with the same specific FA gene mutation site. The most frequent mutation detected was the N991D mutation of the BRCA2 gene, found in four samples. Therefore, we categorized the cases into wild-type and mutant FA groups for further analysis. Due to the low frequency of mutations in the FA gene, we did not filter the mutation sites. We objectively presented the specific mutation sites of the FA gene in the protein structure without classifying the variants as benign or malignant. A preliminary analysis was conducted to evaluate the clinical significance of the mutation sites by utilizing publicly accessible ClinVar databases. Most of the identified variants were categorized as Benign/Likely benign or of uncertain significance. For example, the N991D variant of the BRCA2 gene was identified as benign, while the I917V variant of FANCM was classified as benign/likely benign. Nevertheless, the clinical significance of the R163H variant of the FANCA gene remains undetermined.

Interestingly, the FA mutation group exhibited a more favorable clinical overall survival prognosis in patients with MDS/AML, MDS, or AML. This observation could be attributed to the increased sensitivity of tumor cells carrying FA mutations to chemotherapy drugs. Furthermore, a higher TMB was closely associated with the response to immunotherapy in cancer cases, including AML [30, 31]. Notably, the FA Mut group demonstrated a higher TMB value than the FA Wt group. During stressful conditions, FA genes maintain genomic integrity within cells by participating in DNA repair [1, 32]. The mutation of FA genes may affect the DNA repair of normal cells, thereby contributing to the development of tumors. However, the complex mechanism of tumorigenesis involves the malfunction of various DNA repair proteins. In FA patients with FA mutations, the dysfunctional hemocytes are prone to death under stressful conditions, suggesting that tumor cells possess impaired DNA repair function and face challenges in rapid repair, consequently leading to a more favorable drug response. This partially elucidates the better OS prognosis observed in the FA mutation group.

Pediatric patients with FA and AML have been reported to benefit from Azacitidine [33]. When sufficient data becomes available, it is worthwhile to investigate the mechanisms through which FA transforms into tumors and the chemosensitivity profiles for FA-related genes. FA primarily affects children [1–5], and leukemia development in FA children is associated with a poor survival rate [34]. In this study, we also observed a significantly increased proportion of MDS/AML patients aged 4 to 10 years in the FA mutation group compared to the FA wild-type group. Pediatric MDS/AML patients show a more robust functional correlation with FA pathway genes, which warrants additional attention.

It is essential to determine the treatment plan for AML patients based on cytogenetic risk [35]. Our findings showed that AML patients in the intermediate and poor cytogenetic risk groups have a worse prognosis than those in the favorable group. To predict cytogenetic risk, we developed a random forest classifier incorporating FAB, FANCI, FAAP100 expression, age, and other factors. We also constructed a decision tree using FAB, FLT3 mutation, NPM1 mutation, FANCI, and PABL3 gene expression and analyzed their relationship with cytogenetic risk. Additionally, by considering gender, race, age, FAB, FA/NPM1/FLT3 mutation, and cytogenetic risk, we created a Nomogram to predict the AML survival rates. These findings provide valuable insights into assessing the prognosis of MDS/AML patients according to the expression and mutation patterns of FA signaling pathway members.

The limited prevalence of individuals affected by rare diseases and rare mutations contributes to the paucity of available data. Our study has compiled an extensive dataset encompassing more than 4000 patients diagnosed with AML and MDS, explicitly focusing on FA mutations and, notably, the expression patterns of FA genes. Nevertheless, it is crucial to acknowledge that many patients lack information regarding their FA status and prognostic indicators. As a result, the inadequate sample size for specific subgroup analyses and the omission of additional prognostic assessments hindered a comprehensive evaluation of the association between FA expression, mutation, and prognosis of AML-MDS. To achieve a more impartial assessment, expanding the sample size to encompass a wider spectrum of clinical prognosis and survival outcomes is crucial.

## Conclusions

In summary, this study analyzes data collected from 4259 AML/MDS patients across 10 cohorts. We investigated the mutation spectrum and expression profile of FA signaling pathway genes, and examined the correlation between the FA gene expression/mutations and the survival prognosis of AML/MDS patients. Additionally, a Nomogram has been developed to predict the overall survival rates of AML patients. Moreover, an efficient FA-related random forest/decision tree classifier has been established to assess the cytogenetic risk of AML.

### Supplementary Information

**Additional file 1:** **Fig. S1.** Mutation feature of FA pathway genes with relatively low mutation frequency in cases of MDS/AML. (a) RAD51; (b) UBE2T; (c) RAD51C; (d) RFWD3; (e) FAAP100; (f) FANCE; (g) FANCC; (h) BRIP1; (i) FANCL. **Fig. S2.** Heat map data. We employed the “pheatmap” R package to visually represent the correlation between the mutation, expression, and relevant clinical traits of FA pathway genes. **Fig. S3.** Correlation between FA pathway gene expression and FAB. The expression pattern of FA pathway genes in different FAB groups was visualized using the “ggballoonplot” function. **Fig. S4.** KM survival curve analysis of FA gene expression. Based on the expression matrix of the FA gene, the R packages of “survival” and “survminer” were used for the overall survival prognosis analysis. The “surv_cutpoint” function selected the optimal cutoff. **Fig. S5.** Prognosis evaluation of FA mutation and cytogenetic risk. Based on FA mutation and cytogenetic risk factors, we performed the overall survival prognosis analysis of AML patients using the R packages of “survival” and “survminer”. (a) overall AML patients; (b) favorable risk subgroup; (c) intermediate-risk subgroup; (d) poor-risk subgroup. **Fig. S6.** Prognosis evaluation of FA mutation and TMB. Based on FA mutation and TMB factors, we performed the overall survival prognosis analysis of AML patients, using the R packages of “survival” and “survminer”. (a) MDS + AML; (b) MDS; (c) AML; (d) subgroup analyses of AML patients with or without FA mutation. **Fig. S7.** Prognosis evaluation of FLT3/FA mutation. Based on FLT3 and FA mutation factors, we performed the overall survival prognosis analysis of AML patients, using the R packages of “survival” and “survminer”. (a) MDS + AML; (b) AML; (c) subgroup analyses of AML patients with or without FA mutation. **Fig. S8.** Prognosis evaluation of NPM1/FA mutation. Based on the factors of NPM1 mutation and FA mutation, we performed the overall survival prognosis analysis of AML patients, using the R packages of “survival” and “survminer”. (a) AML; (b) MDS + AML; (c) subgroup analyses of AML patients with or without FA mutation.

## Data Availability

The datasets used and analyzed during the current study are available from the corresponding author upon reasonable request.

## Abbreviations

FA: Fanconi anemia
MDS: myelodysplastic syndromes
AML: acute myeloid leukemia
PCA: principal component analysis
ROC: receiver operating characteristic curve
AUC: the area under the ROC curve
CI: confidence interval
FAB: French-American-British
TMB: tumor mutational burden
