# Supplementary figures and images for: Mutation spectrum, expression profiling, and prognosis evaluation of Fanconi anemia signaling pathway genes for 4259 patients with myelodysplastic syndromes or acute myeloid leukemia
Source: BMC Med Genomics. 2023 Nov 16;16:290. doi: 10.1186/s12920-023-01730-5 (PMC10652513; doi:10.1186/s12920-023-01730-5)

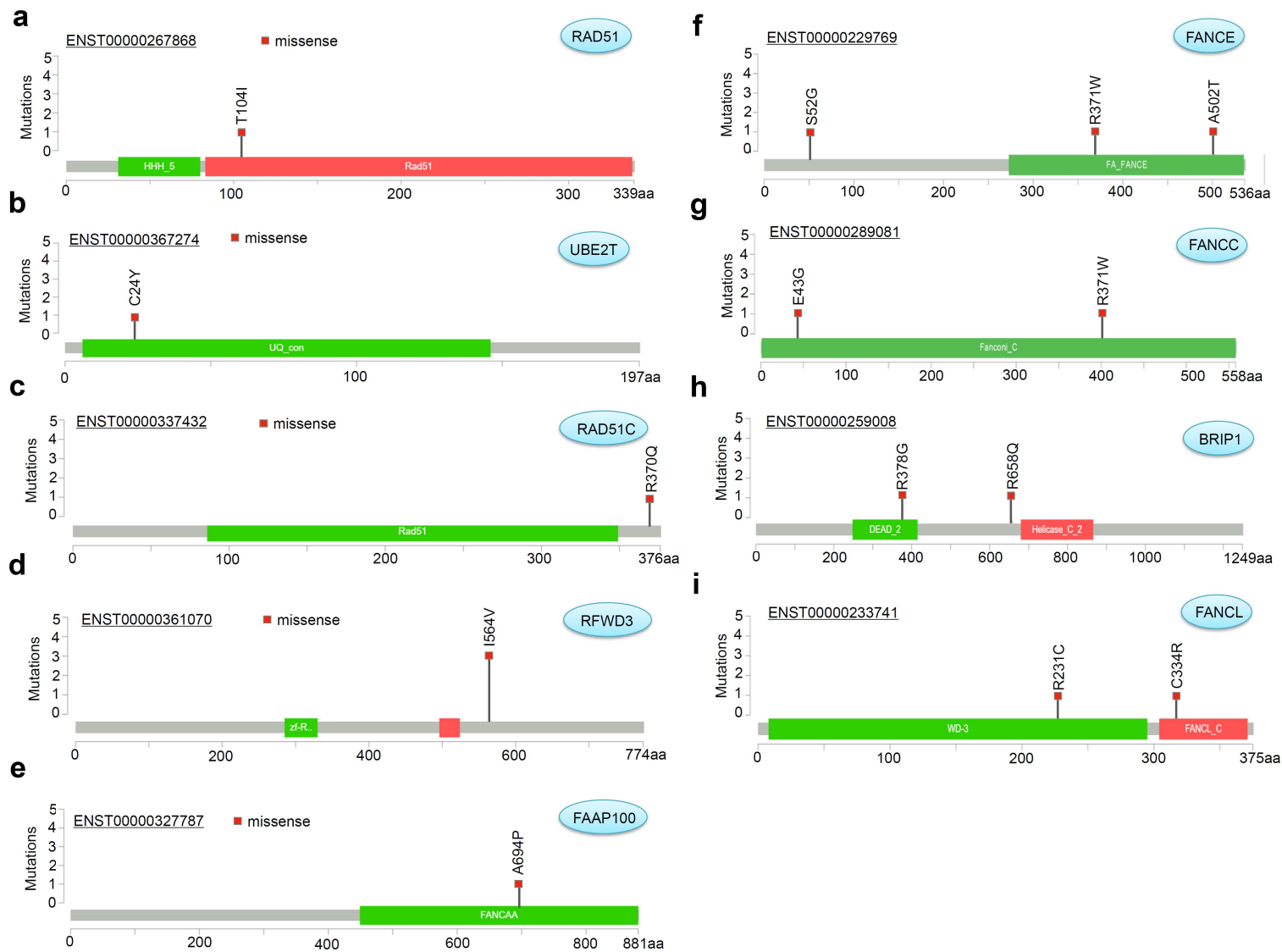

**Figure S1**

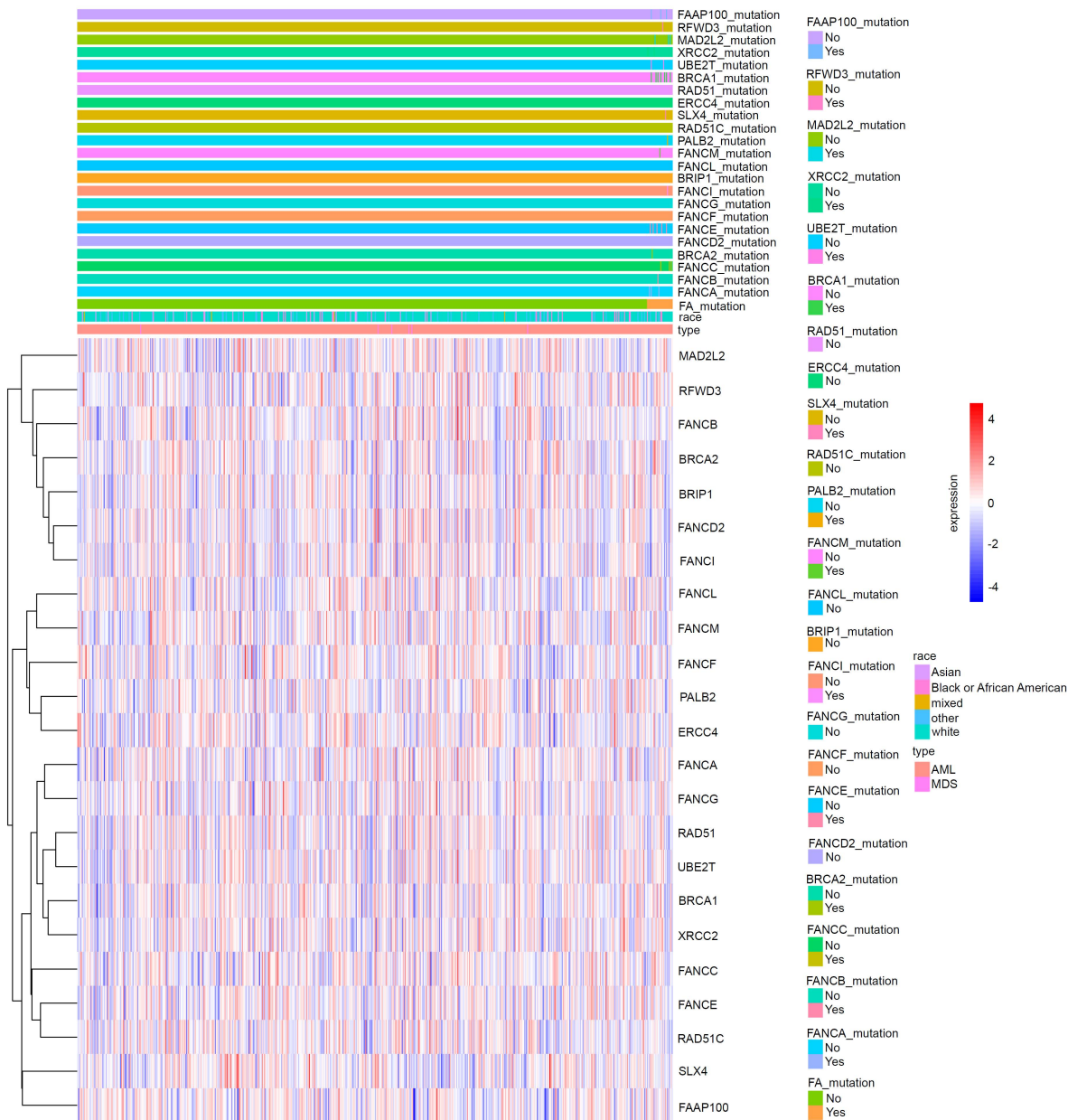

**Figure S2**

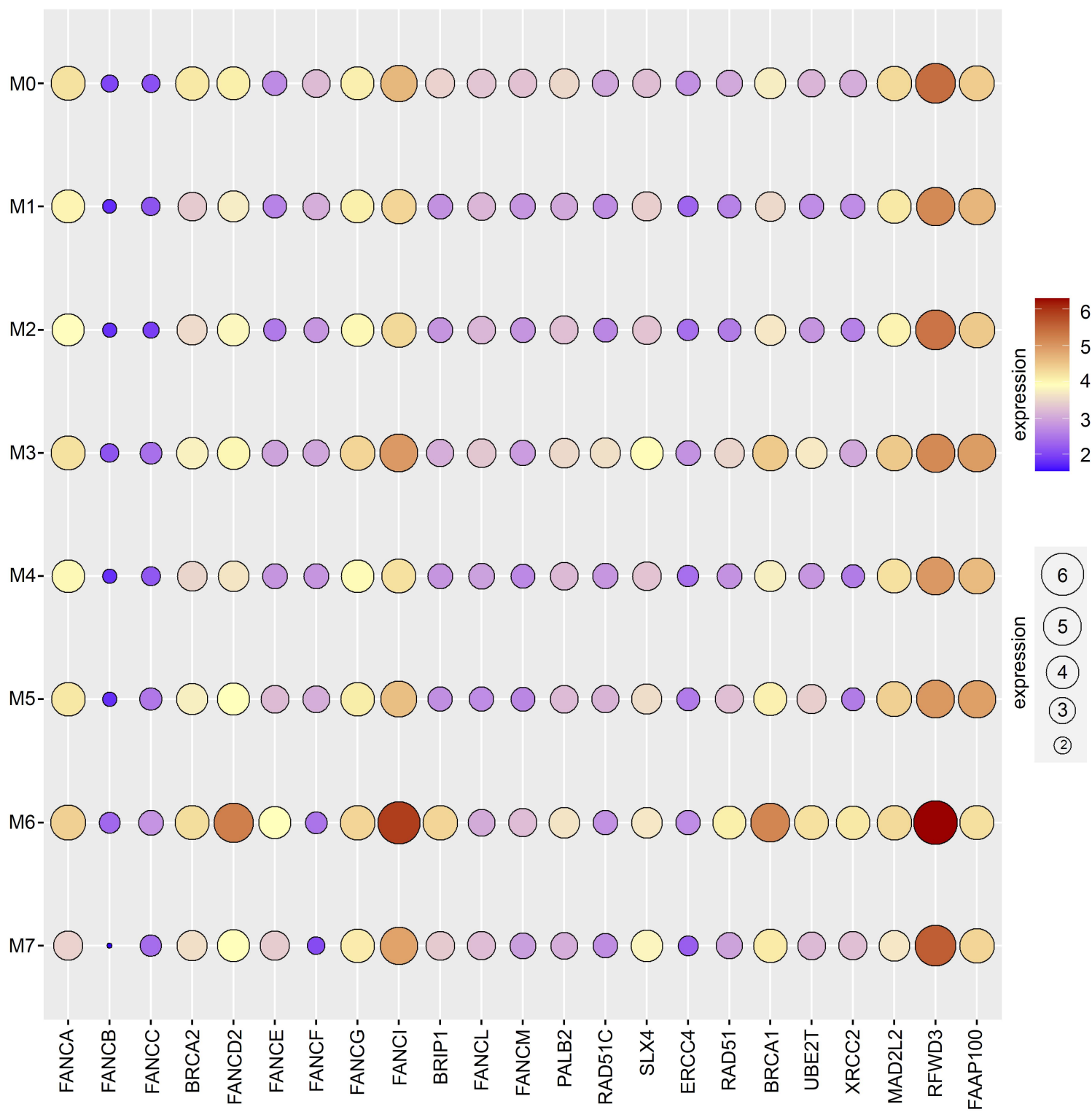

**Figure S3**

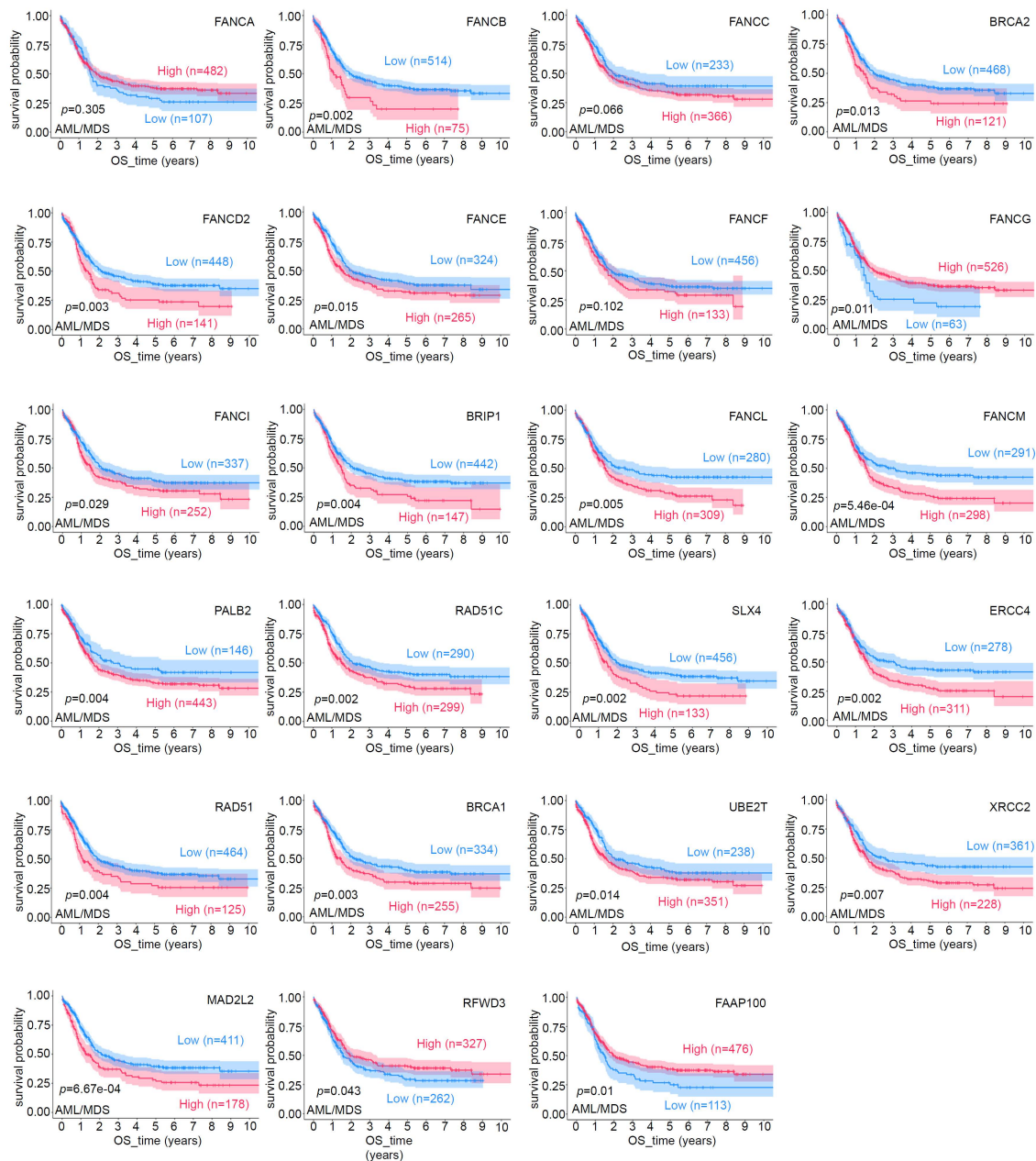

**Figure S4**

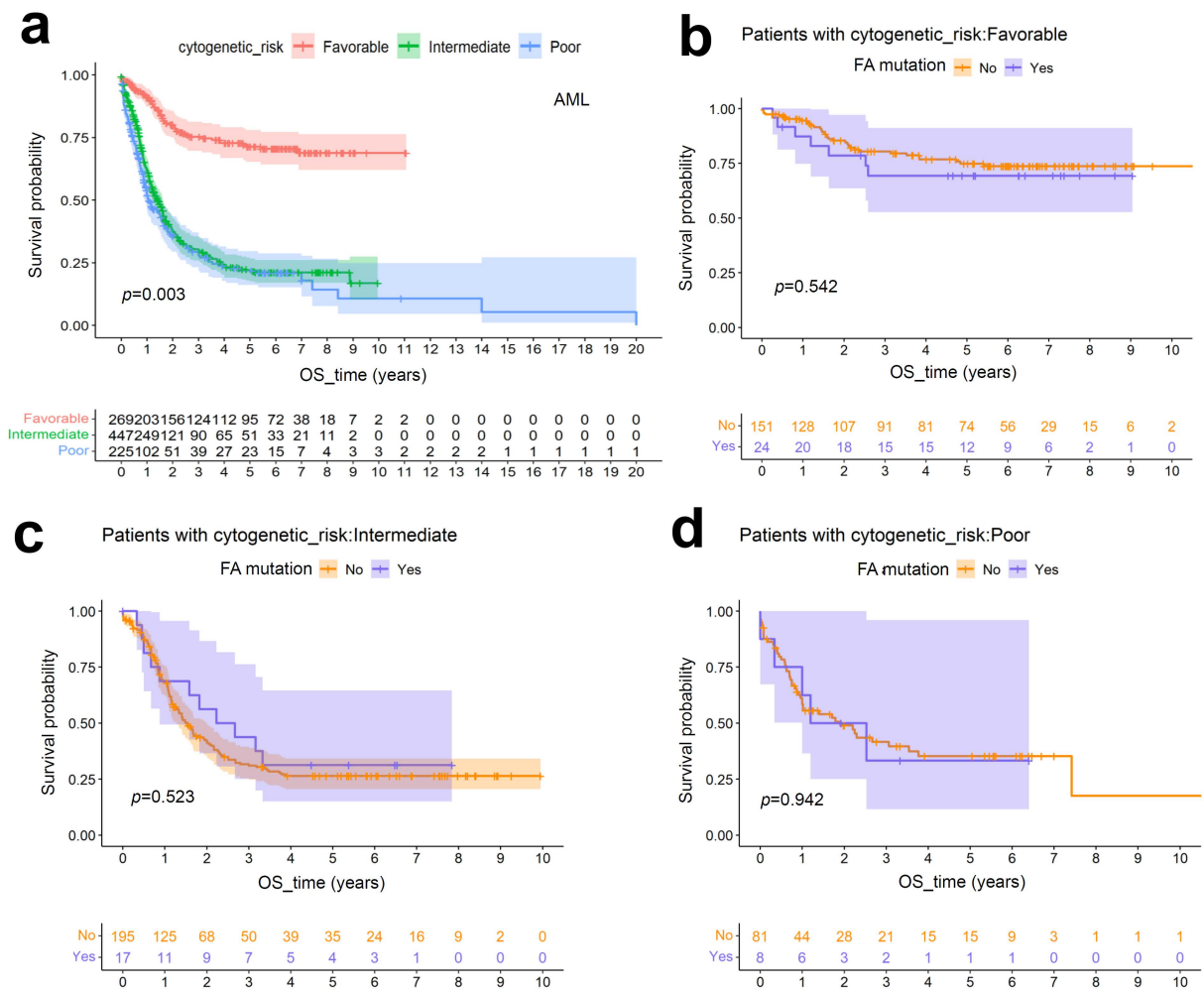

**Figure S5**

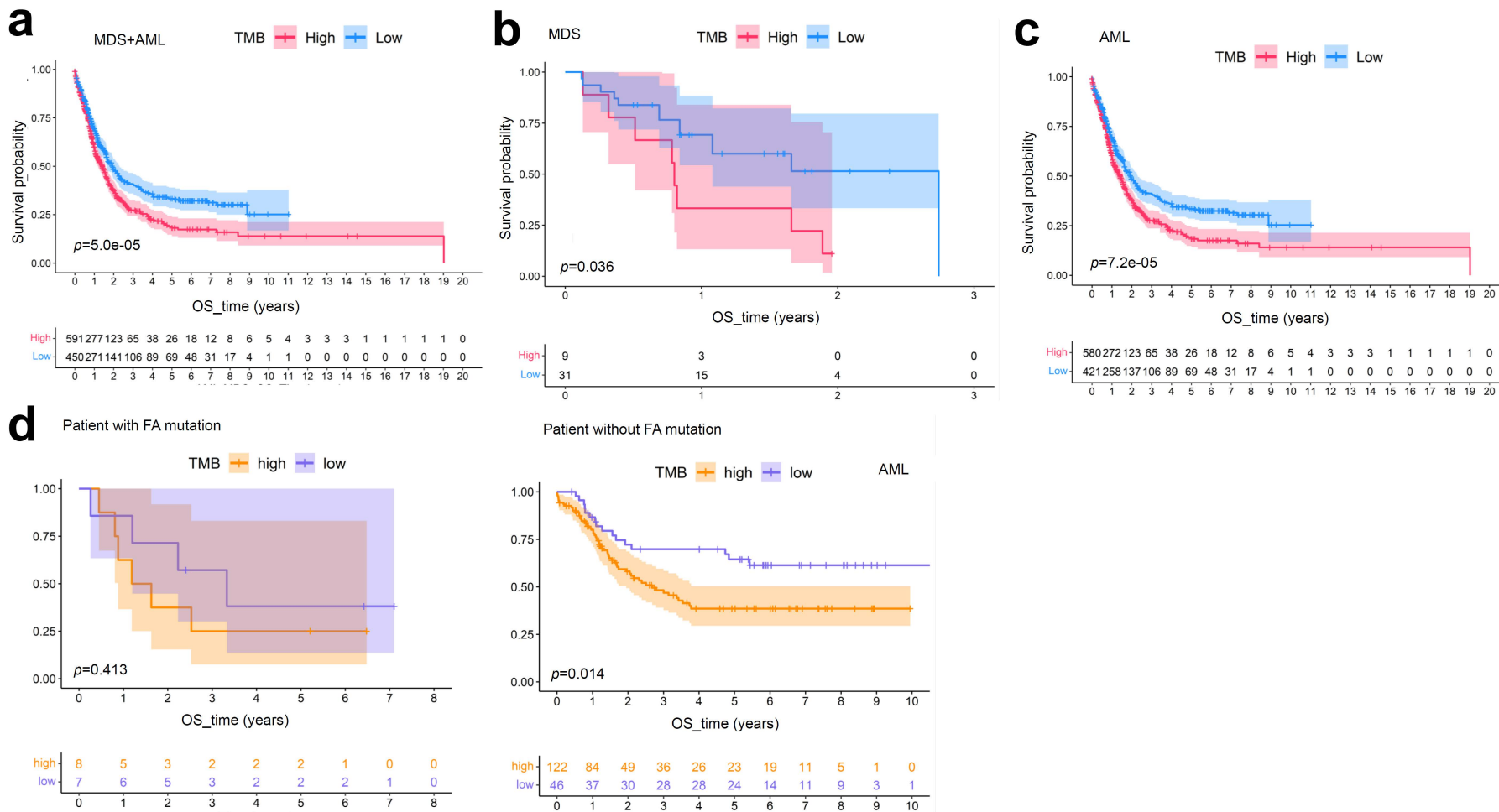

**Figure S6**

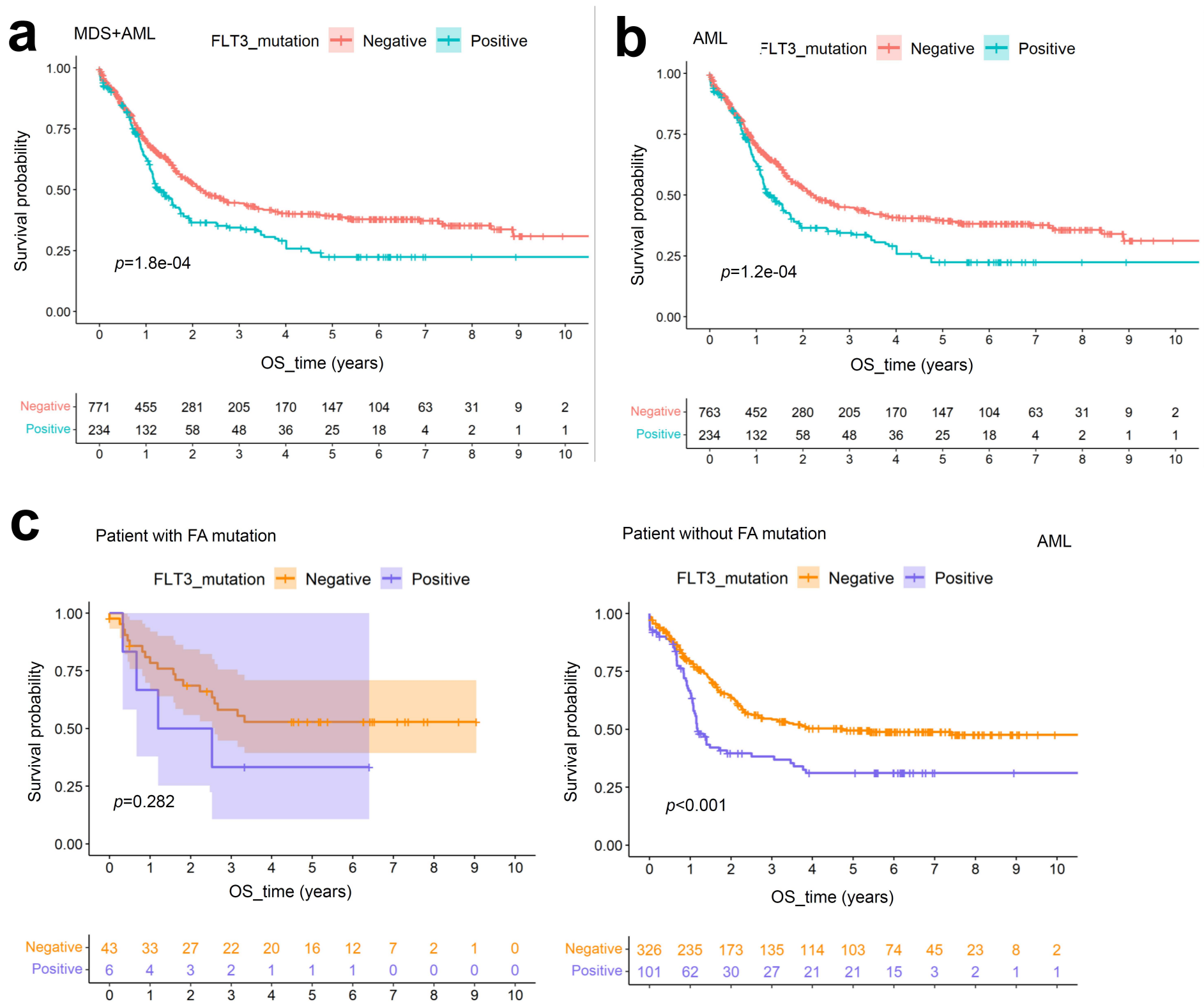

**Figure S7**

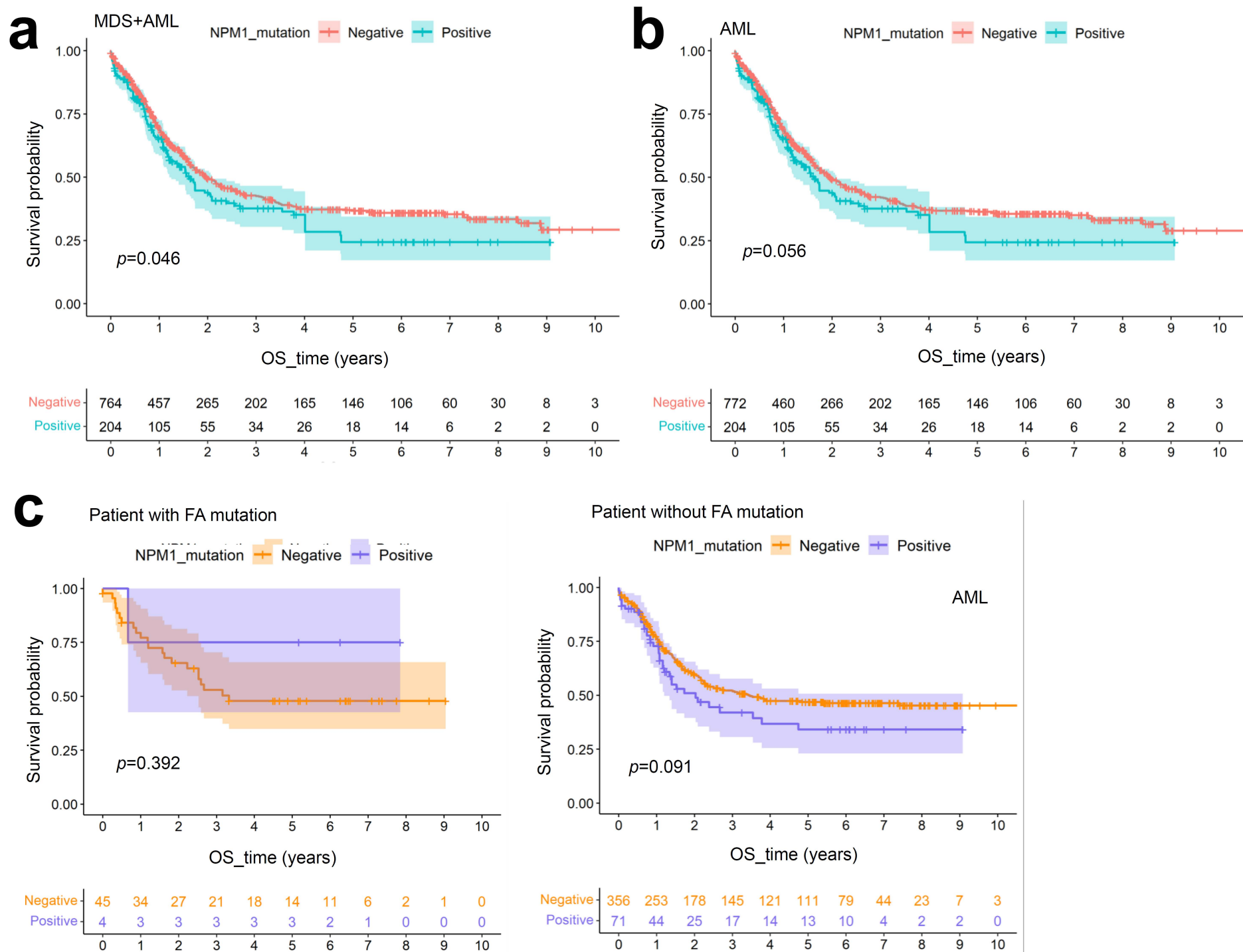

**Figure S8**

Supplement: Supplementary file 1 — Additional file 1: Fig. S1. Mutation feature of FA pathway genes with relatively low mutation frequency in cases of MDS/AML. (a) RAD51; (b) UBE2T; (c) RAD51C; (d) RFWD3; (e) FAAP100; (f) FANCE; (g) FANCC; (h) BRIP1; (i) FANCL. Fig. S2. Heat map data. We employed the “pheatmap” R package to visually represent the correlation between the mutation, expression, and relevant clinical traits of FA pathway genes. Fig. S3. Correlation between FA pathway gene expression and FAB. The expression pattern of FA pathway genes in different FAB groups was visualized using the “ggballoonplot” function. Fig. S4. KM survival curve analysis of FA gene expression. Based on the expression matrix of the FA gene, the R packages of “survival” and “survminer” were used for the overall survival prognosis analysis. The “surv_cutpoint” function selected the optimal cutoff. Fig. S5. Prognosis evaluation of FA mutation and cytogenetic risk. Based on FA mutation and cytogenetic risk factors, we performed the overall survival prognosis analysis of AML patients using the R packages of “survival” and “survminer”. (a) overall AML patients; (b) favorable risk subgroup; (c) intermediate-risk subgroup; (d) poor-risk subgroup. Fig. S6. Prognosis evaluation of FA mutation and TMB. Based on FA mutation and TMB factors, we performed the overall survival prognosis analysis of AML patients, using the R packages of “survival” and “survminer”. (a) MDS + AML; (b) MDS; (c) AML; (d) subgroup analyses of AML patients with or without FA mutation. Fig. S7. Prognosis evaluation of FLT3/FA mutation. Based on FLT3 and FA mutation factors, we performed the overall survival prognosis analysis of AML patients, using the R packages of “survival” and “survminer”. (a) MDS + AML; (b) AML; (c) subgroup analyses of AML patients with or without FA mutation. Fig. S8. Prognosis evaluation of NPM1/FA mutation. Based on the factors of NPM1 mutation and FA mutation, we performed the overall survival prognosis analysis of AML patients, [file 12920_2023_1730_MOESM1_ESM.pdf]
